# Physiological responses to food intake throughout the day

**DOI:** 10.1017/S0954422414000055

**Published:** 2014-06

**Authors:** Jonathan D. Johnston

**Affiliations:** Faculty of Health and Medical Sciences, University of Surrey, Guildford, SurreyGU2 7XH, UK

**Keywords:** Circadian clock, Metabolism, Breakfast, Night eating syndrome

## Abstract

Circadian rhythms act to optimise many aspects of our biology and thereby ensure that physiological processes are occurring at the most appropriate time. The importance of this temporal control is demonstrated by the strong associations between circadian disruption, morbidity and disease pathology. There is now a wealth of evidence linking the circadian timing system to metabolic physiology and nutrition. Relationships between these processes are often reciprocal, such that the circadian system drives temporal changes in metabolic pathways and changes in metabolic/nutritional status alter core molecular components of circadian rhythms. Examples of metabolic rhythms include daily changes in glucose homeostasis, insulin sensitivity and postprandial response. Time of day alters lipid and glucose profiles following individual meals whereas, over a longer time scale, meal timing regulates adiposity and body weight; these changes may occur via the ability of timed feeding to synchronise local circadian rhythms in metabolically active tissues. Much of the work in this research field has utilised animal and cellular model systems. Although these studies are highly informative and persuasive, there is a largely unmet need to translate basic biological data to humans. The results of such translational studies may open up possibilities for using timed dietary manipulations to help restore circadian synchrony and downstream physiology. Given the large number of individuals with disrupted rhythms due to, for example, shift work, jet-lag, sleep disorders and blindness, such dietary manipulations could provide widespread improvements in health and also economic performance.

## Introduction

Circadian rhythms are cyclical endogenous processes that occur with a periodicity of approximately 24 h. They are found throughout the natural world, from simple unicellular organisms through to human beings^(^ ^1^ ^)^. Possession of such rhythms enables organisms to anticipate predictable changes in the environment and thus adapt their physiology accordingly. Temporal control over metabolic processes also allows cells and organisms to separate opposing biochemical pathways, for example, redox reactions and anabolism *v.* catabolism. Moreover, in model species, it has been demonstrated that possession of circadian rhythms that synchronise to environmental changes confers a selective advantage^(^ ^2^ ^)^.

A great deal of current research is being undertaken at the interface between the circadian timing system, metabolic physiology and nutritional science. Studying how these major biomedical areas inter-relate will not only increase our understanding of healthy metabolism, but may also guide the development of nutritional interventions for body-weight regulation, the management of obesity-related disease and the treatment of circadian disorders associated with shift work, jet-lag, abnormal sleep phase and blindness.

## The mammalian circadian timing system

It was recognised over 40 years ago that a small brain region within the anterior hypothalamus, the suprachiasmatic nuclei (SCN), is important for the expression of circadian rhythms in mammals^(^ ^3^ ^,^ ^4^ ^)^. When the SCN are isolated from surrounding brain tissue *in vivo*, or maintained as tissue explants *in vitro*, their neurones maintain robust rhythmicity^(^ ^5^ ^–^ ^7^ ^)^. Furthermore, if SCN tissue from one animal is transplanted to another animal that has had its SCN lesioned, the resulting behavioural rhythms reflect that of the donor animal, not the host^(^ ^8^ ^)^. It is therefore clear that the SCN play a key role in the generation of mammalian circadian rhythms.

Mammalian clocks outside the SCN, termed ‘peripheral clocks’, were first identified in tissues such as the retina, which exhibits rhythmic hormone secretion when maintained in culture^(^ ^9^ ^)^. Following the cloning of genetic components of the mammalian clock came the discovery of rhythmic clock gene expression in peripheral tissues^(^ ^10^ ^,^ ^11^ ^)^. Subsequent advances came from the use of transgenic animals in which reporter gene expression is driven by clock gene elements. Real-time imaging of tissue explants taken from these animals confirmed that many peripheral tissues possess an endogenous clock^(^ ^12^ ^,^ ^13^ ^)^. Perhaps most surprisingly, circadian rhythms have also been identified in cultures of immortalised cells^(^ ^14^ ^–^ ^16^ ^)^.

In animal models, rhythmic clock gene expression is known to occur in multiple tissues involved in metabolism and nutritional physiology, including the liver, pancreas, gastrointestinal tract, adipose tissue and skeletal muscle^(^ ^17^ ^,^ ^18^ ^)^. For ethical and technical reasons, molecular analysis of human tissues is difficult and various strategies have been adopted to study human clock gene expression^(^ ^19^ ^)^. However, clock gene rhythms have now successfully been observed in human leucocytes^(^ ^20^ ^,^ ^21^ ^)^, fibroblasts^(^ ^22^ ^,^ ^23^ ^)^ and adipose tissue^(^ ^24^ ^,^ ^25^ ^)^, with single time point analysis of clock gene expression in other tissues, including pancreatic islets^(^ ^26^ ^)^.

The presence of rhythms throughout the body requires appropriate physiological mechanisms to keep tissues correctly synchronised to one another. The SCN receive photic information directly from the retina and are readily synchronised to the external light–dark cycle^(^ ^27^ ^)^. In normal circumstances, the clock in the SCN then synchronises rhythms elsewhere in the body through a variety of output pathways^(^ ^28^ ^)^. A commonly used analogy to describe this organisation refers to the SCN as a conductor of an orchestra, with the peripheral tissues representing individual musicians; each of the ‘musicians’ is capable of generating its own time but requires the central ‘conductor’ to ensure that they all maintain correct time relative to each other and thus optimal overall output.

There are many ways through which the SCN can synchronise peripheral tissues. These include endocrine and neuronal pathways, such as the secretion of glucocorticoids and the tone of the autonomic nervous system^(^ ^28^ ^)^. In addition, by influencing the timing of sleep–wake rhythms, the SCN also dictate the timing of certain behaviours, for example, feeding, which are thought to be critical to the rhythms in peripheral tissues as explained below.

## Metabolic functions of circadian timing and specific roles of peripheral clocks

Since the identification of clocks in peripheral tissues, a critical challenge has been to identify their physiological role. An early indication that peripheral clocks had a strong influence on metabolism came from transcriptomic analyses. Depending on the analytical methods used, these studies estimated that up to 20 % of the transcriptome in peripheral tissues exhibits 24 h variation^(^ ^29^ ^–^ ^33^ ^)^. Identification of the rhythmic transcripts revealed a large cluster of genes encoding proteins involved in metabolic pathways. Later proteomic analysis also suggested that up to 20 % of proteins in the mouse liver oscillate with a circadian rhythm, and many of these proteins are indeed involved in important metabolic functions^(^ ^34^ ^)^. Technical advances have since permitted direct analysis of the daily metabolome in different tissues. Similar to the transcriptomic and proteomic data, both mouse^(^ ^35^ ^–^ ^37^ ^)^ and human^(^ ^38^ ^–^ ^40^ ^)^ studies estimate that up to 20 % of the metabolome is under 24 h regulation.

Genetic evidence for a role of circadian clocks in key metabolic processes is now substantial. As discussed previously^(^ ^41^ ^)^, the precise nature of metabolic abnormality in transgenic animals depends upon their genetic background. Nonetheless the dysregulation of key metabolic processes, including glucose and lipid homeostasis, following disruption of key genes involved in circadian biology reveals fundamental links between circadian genetics and metabolism^(^ ^42^ ^–^ ^47^ ^)^. Consistent with these animal data, a number of groups have now reported correlations between aspects of human metabolism and clock gene polymorphisms^(^ ^48^ ^–^ ^53^ ^)^.

One limitation of studies involving individuals with ‘whole body’ genetic changes is that they do not clearly indicate the contribution of individual tissue rhythms to whole-organism physiology. Using the Cre-*Lox* recombinase system to disrupt the *Bmal1* (brain and muscle arnt-like protein-1) gene in a tissue-specific manner, circadian rhythms in the liver, pancreas and white adipose tissue have been selectively ‘knocked out’ allowing the *in vivo* role of their clocks to be investigated. Mice bearing a liver-specific clock disruption exhibit increased glucose clearance following acute challenge, fasting hypoglycaemia and other features suggesting that the hepatic clock regulates glucose export into the blood^(^ ^54^ ^)^. By contrast, disruption of the pancreatic clock results in hyperglycaemia, reduced glucose tolerance and impaired insulin secretion^(^ ^55^ ^,^ ^56^ ^)^. Finally, knock-out of *Bmal1* in white adipose tissue induces obesity; a contributory mechanism to this phenotype is the temporal modification of PUFA signalling from adipocytes to appetite-regulatory regions of the hypothalamus, leading to increased feeding during the resting phase of the day^(^ ^57^ ^)^. Thus, circadian dysfunction in individual tissues can lead to major changes in whole-body energy metabolism.

## Timed feeding as a synchroniser of peripheral clocks

### The food-entrainable oscillator

Restriction of food availability to a narrow time window each day results in profound reorganisation of behaviour and physiology^(^ ^58^ ^,^ ^59^ ^)^. Such temporal restriction induces a bout of activity in advance of food availability, termed ‘food anticipatory activity’ (FAA). This phenomenon was originally observed in rats, but has since been reported in multiple vertebrate and invertebrate species. More detailed analysis in rodents reveals that this FAA is also accompanied by physiological changes including increased core body temperature and serum glucocorticoid concentration.

Interestingly, FAA exhibits properties that are consistent with it being controlled by endogenous circadian clock(s), rather than being merely a food-driven phenomenon. For example, if the temporal window of food availability is abruptly delayed, the onset of FAA takes multiple 24 h cycles to resynchronise to the new feeding time. Moreover, if an animal is completely food deprived, FAA persists at approximately the same time every 24 h for as long as the food deprivation can be maintained^(^ ^58^ ^,^ ^59^ ^)^.

The underlying circadian basis of FAA has led to the postulation that animals contain a food-entrainable oscillator (FEO). Although there are reported differences in FAA in mice lacking genetic components of the circadian clock^(^ ^60^ ^)^, these mice do retain the ability to display FAA^(^ ^61^ ^)^. It is therefore believed that the genetic control of the FEO differs from other circadian processes. In keeping with this idea, food anticipatory responses persist in SCN-lesioned animals^(^ ^62^ ^,^ ^63^ ^)^, indicating that the FEO resides in tissue(s) outside of the SCN, the master circadian clock. Some studies have suggested that the FEO may be closely linked to the dorsomedial hypothalamic nuclei, a brain region known to be involved in the homeostatic regulation of feeding^(^ ^64^ ^,^ ^65^ ^)^. Other work has highlighted the potential role of extra-hypothalamic brain regions in FAA^(^ ^66^ ^)^. The anatomical localisation of the FEO remains a controversial topic, however^(^ ^67^ ^–^ ^69^ ^)^. Indeed the FEO may lie outside of the brain or require the interplay between multiple tissues.

### Regulation of rhythms in peripheral tissues

One potential mechanism underlying food-entrainable rhythmicity is the effect of feeding time on peripheral tissue clocks. In normal physiological conditions, the timing of behavioural rhythms, such as feeding, is driven by the SCN and thus represents a mechanism through which the SCN can synchronise rhythms in the periphery. The powerful nature of timed feeding as a circadian signal becomes apparent when food availability is divorced from SCN rhythms.

**Fig. 1:**
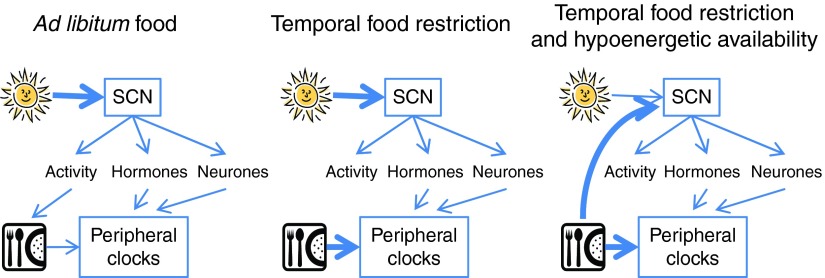
Regulation of the circadian timing system by light and food. Under normal conditions of *ad libitum* food, light synchronises the master clock, the suprachiasmatic nuclei (SCN), which then synchronises peripheral clocks via neuronal and endocrine pathways, together with control over behavioural activity and thus feeding time. When feeding time (but not energy availability) is restricted, light remains the dominant synchroniser of the SCN, but peripheral clocks are synchronised to feeding time. Under conditions of temporal and energy food restriction, both the SCN and peripheral clocks are synchronised to the feeding time. (A colour version of this figure can be found online at http://www.journals.cambridge.org/nrr)

A typical protocol restricts food availability to nocturnal rodents, so that they can only eat during the light period of a light–dark cycle. This inverts the phase of clock gene rhythms in peripheral tissues, such as the liver, kidney, heart, pancreas, lung, gastrointestinal tract, and brown and white adipose tissue^(^ ^33^ ^,^ ^70^ ^–^ ^72^ ^)^. Full entrainment of the liver rhythms appears to occur within 2–3 d, whereas the other peripheral tissues may require up to 1 week before they exhibit the maximal phase shift. A later mouse study involved removal of food access for the first 6 h of the dark period of a light–dark cycle, with mild energy restriction. After 4 d in this protocol, mice exhibited delayed rhythms of hepatic and plasma TAG concentration, together with delayed rhythms of lipogenic and clock gene expression in both liver and adipose tissue^(^ ^73^ ^)^. When animals are able to eat *ad libitum* quantities of food during temporal restriction paradigms, SCN rhythms remain locked to the light–dark cycle^(^ ^70^ ^,^ ^71^ ^)^. However, the combination of temporal food restriction and hypoenergetic food availability does induce reorganisation of rodent SCN rhythms^(^ ^74^ ^,^ ^75^ ^)^. Indeed, hypoenergetic feeding of nocturnal rodents without restricting food availability to the light period alters the phase of SCN-driven rhythms^(^ ^76^ ^)^ as well as gene expression in peripheral tissues^(^ ^77^ ^)^. Thus the overall effect of feeding on circadian organisation appears to involve an interaction between both the timing and the quantity of food intake (Fig. 1).

An important caveat when extrapolating the studies described above to human society is the relevance to standard human meal patterns. A common theme in human society is a feeding pattern of three meals per d. In contrast, most animal studies to date have utilised prolonged *ad libitum* feeding opportunities restricted to certain phases of the 24 h day. Some studies, however, have developed a ‘humanised’ meal protocol for rodents. When rats are only given their daily energy intake over ‘lunch’ and ‘dinner’ their gene expression rhythms in liver, heart and white adipose tissue are delayed compared with a group receiving the same total energy intake spread over three meals^(^ ^78^ ^)^. Consistent with this finding, studies in mice using a range of mealtime combinations indicate that the first meal following a long fasting period provides an important synchronising signal to peripheral clocks^(^ ^77^ ^,^ ^79^ ^)^. To date there are no comparable molecular data from human studies. One rare investigation of timed feeding on human circadian physiology is an experiment in which subjects were fed a single daily carbohydrate-rich meal for 3 d; morning consumption of this meal advanced core body temperature and heart rate, but not melatonin, rhythms compared with evening meal timing^(^ ^80^ ^)^. Furthermore, a delay in the timing of three daily meals within a fixed light–dark cycle is known to delay the phase of plasma leptin rhythms^(^ ^81^ ^)^, which may be at least partially due to changes in adipose tissue clocks.

Although redundancy of signalling pathways providing input to the relevant tissues hinders elucidation of mechanistic insight into food entrainment, some progress has been made. At the nutritional level, phase shifting of the liver clock seems greater when the starch component of a mixed diet provides a large postprandial glucose concentration^(^ ^82^ ^)^. Interestingly, however, ingestion of 100 % glucose, sucrose or maize starch is insufficient to alter the phase of liver rhythms, indicating that mixed macronutrient content may be necessary for food entrainment in the liver^(^ ^83^ ^)^. At the physiological level, temporal food restriction more rapidly resynchronises peripheral clock gene rhythms in mice that have been adrenalectomised, compared with sham-operated controls^(^ ^84^ ^)^. This finding suggests that glucocorticoid signalling, which is believed to be an endocrine link between the SCN and peripheral clocks^(^ ^85^ ^)^, may inhibit or delay the impact of temporal food availability. At the molecular level, it has been demonstrated that rhythmic phosphorylation of key transcription factors CREB and Akt (cAMP response element-binding protein and protein kinase B) in the mouse liver is driven by temporal feeding patterns^(^ ^86^ ^)^. In keeping with this result, the expression of multiple genes targeted by molecular nutrient and stress sensors was similarly dependent on feeding time^(^ ^86^ ^)^. Finally, mice deficient for the gene *Parp1* (poly [ADP-ribose] polymerase 1) or the neurone-specific γ form of protein kinase C (PKCγ) exhibit impaired synchronisation to timed feeding^(^ ^87^ ^,^ ^88^ ^)^. Despite these advances, the mechanisms that mediate the effects of food on the circadian system are poorly understood and will doubtless be the subject of multiple further studies.

## Human metabolic physiology and postprandial responses vary across the day

### Diurnal changes

Diurnal rhythmicity refers to 24 h changes that occur in individuals kept in a varying environment, for example, a 24 h light–dark cycle. Although such rhythms are often relevant to a real-life scenario, there is the possibility that they are driven by environmental fluctuations rather than endogenous processes *per se*. As a result, they are not considered to be truly circadian.

Arguably the best-characterised daily metabolic rhythms in humans relate to changes in glucose homeostasis. Diurnal changes in glucose tolerance have been recognised in human subjects for many years^(^ ^89^ ^)^. Sensitivity to elevated glucose concentration is greatest in the early morning and then declines over the course of the day, leading to a phenomenon that has been termed ‘afternoon diabetes’. This daily change is not dependent upon changes in gastrointestinal function, but instead appears to be the result of altered glucose utilisation and insulin sensitivity, with maximal insulin sensitivity occurring in the early morning and decreasing throughout the day^(^ ^89^ ^)^.

In addition to glucose homeostasis, the regulation of plasma lipids is also subject to daily variation. Not only are basal concentrations of TAG elevated at night, but also there are diurnal changes in the postprandial TAG response. Ingestion of a meal at night results in increased plasma TAG that remains elevated for longer than the response to the same meal given during the day^(^ ^90^ ^)^. A study of postprandial responses to breakfast and lunch reported approximately 50 % less change in plasma TAG concentration following lunch than breakfast, despite the plasma TAG fraction showing no differences in concentration of [^13^C]palmitic acid that was included in each meal^(^ ^91^ ^)^. The physiological basis for temporal differences in postprandial TAG response may therefore be independent of absorption or mobilisation of meal-derived lipids from the gut^(^ ^91^ ^)^.

A number of groups have studied temporal variation of adipokines, which are adipose-derived hormones that regulate metabolic physiology in the brain and multiple peripheral tissues^(^ ^92^ ^,^ ^93^ ^)^. Diurnal rhythms have been reported for many of these hormones, including leptin, adiponectin, chemerin, lipocalin and visfatin^(^ ^94^ ^–^ ^98^ ^)^. Although the secretion of these hormones is likely to be governed by multiple factors such as feeding and sleep, detailed analyses of leptin secretion suggest that there is likely to be an underlying circadian component to adipokine rhythmicity^(^ ^99^ ^,^ ^100^ ^)^. Furthermore, given the functional roles of adipokines, their rhythmic secretion may make important contributions towards the daily changes in glucose and lipid homeostasis described above.

### Identification of endogenous circadian rhythms

In order to unmask truly endogenous circadian rhythms from temporal changes in the environment, a number of different laboratory protocols have been developed. The most widely used of these are the constant routine and forced desynchrony protocols^(^ ^101^ ^,^ ^102^ ^)^. In a constant routine, subjects are kept awake in a supine posture in constant dim light, with identical regular (for example, hourly) snacks. Although this protocol effectively removes environmental rhythms, it does result in the development of sleep debt due to the necessity to keep subjects awake. One solution to this problem is to allow subjects to sleep during the dark phase of a light–dark cycle that is sufficiently different from 24 h to permit entrainment of subjects' circadian rhythms; this is the basis of a forced desynchrony protocol. For example, a commonly used variant of this protocol employs a 28 h light–dark cycle that therefore allows subjects to sleep every 28 h, while their circadian rhythms occur (‘free run’) with a frequency of approximately 24 h.

Various research groups have utilised the above protocols to investigate the contribution of the endogenous circadian system to daily rhythms of glucose and lipid metabolism. One study in which 4-hourly meals were administered over a constant routine revealed elevation of both postprandial glucose and TAG during the biological night, especially after high-fat meal intervention over the week preceding the constant routine^(^ ^103^ ^)^. Consistent with this finding, analysis of postprandial responses during a forced desynchrony of 27-h days revealed effects of both circadian time and length of prior wakefulness on glucose and TAG concentration^(^ ^104^ ^)^. By contrast, postprandial insulin responses were regulated by circadian time, but not length of wakefulness. A more recent forced desynchrony protocol kept volunteers on 28-h days, each of which contained four meals: breakfast, lunch, dinner and a snack shortly before bedtime^(^ ^105^ ^)^. It was found that the times during which subjects were awake and eating during their biological night resulted in multiple cardiometabolic changes, including decreased plasma leptin concentration and increased concentrations of both plasma glucose and insulin. In fact, the postprandial responses of some of these healthy subjects during the biological night were equivalent to the responses of a pre-diabetic individual^(^ ^105^ ^)^. In a separate cross-over study using forced desynchrony, volunteers had daily metabolic profiles assessed after both three 21-h days and also three 27-h days. Although there were some differences in response to the 21-h *v.* 27-h days, both schedules disrupted glucose–insulin metabolism, increased carbohydrate oxidation and reduced protein oxidation, but had little or no effect on appetite or energy balance^(^ ^106^ ^)^. The human circadian system therefore exerts clear influence over key aspects of metabolic physiology.

## Effects of body weight on circadian rhythms

The data discussed above describe the interaction between clocks and metabolism on a relatively short-term time frame. Of importance to health is the longer-term relationship between circadian rhythms, metabolic status and body weight. Indeed, many studies in the literature have reported altered daily rhythms in association with factors including altered body weight, presence of metabolic disease and long-term changes in nutrient intake.

Following the demonstration of daily rhythms of plasma leptin in human subjects, it was reported that the percentage amplitude of these rhythms declined in obese individuals^(^ ^94^ ^,^ ^107^ ^,^ ^108^ ^)^. However, not all studies have been able to replicate this finding^(^ ^109^ ^,^ ^110^ ^)^. The reasons for discrepancies are not clear, but may include varied pre-laboratory controls, sex of subjects, extent of obesity (i.e. BMI of 30–35 *v.* 40+ kg/m^2^) and distribution of fat within the obese subjects recruited.

Another hormone that appears to demonstrate correlation between rhythm amplitude, body weight and metabolic health is melatonin. An early report that nocturnal melatonin concentration positively correlates with BMI in insulin-sensitive human subjects^(^ ^111^ ^)^ has been supported by recent data reporting elevated amplitude melatonin rhythms in obese non-diabetic men, although blunted melatonin rhythms are present in weight-matched men with type 2 diabetes^(^ ^110^ ^)^. In addition, nocturnal melatonin concentration correlates with aspects of the metabolic syndrome in women^(^ ^112^ ^)^. The functional relevance of these endocrine data is supported by molecular and genetic evidence for a role of melatonin signalling in metabolic physiology and type 2 diabetes mellitus. Common polymorphisms of the human MT2 melatonin receptor have been associated with impaired glucose homeostasis and type 2 diabetes in multiple populations^(^ ^113^ ^–^ ^116^ ^)^. Although the calculated risk of developing type 2 diabetes is small for these polymorphisms, subsequent work identified additional rare MT2 variants that confer a much higher risk of diabetes and also disrupt melatonin signalling in cell culture experiments^(^ ^117^ ^)^. Evidence from animal models further supports the existence of a physiological link between melatonin, insulin secretion^(^ ^118^ ^)^ and insulin sensitivity^(^ ^119^ ^,^ ^120^ ^)^. When comparing rodent and human data, it should be recognised that elevated melatonin secretion occurs at night in all species, irrespective of whether they are active at night or during the day; therefore direct translation of data relating melatonin to glucose homeostasis from rodents to humans is difficult. Despite this, there is now evidence to support linking low nocturnal melatonin levels in humans, estimated from morning urinary metabolite concentration, with the risk of developing type 2 diabetes^(^ ^121^ ^)^ and also insulin resistance in non-diabetic subjects^(^ ^122^ ^)^.

Comparison of molecular rhythms in lean and obese individuals has also been addressed by a number of research groups. Reduced amplitude rhythms have been reported in tissues such as adipose^(^ ^123^ ^,^ ^124^ ^)^, liver^(^ ^124^ ^,^ ^125^ ^)^ and brain stem^(^ ^126^ ^)^ of obese and diabetic mice. However, interpretation of these data is sometimes hampered by the use of different genetic strains in the lean and obese groups. We have recently compared daily profiles of clock gene expression in subcutaneous adipose biopsies taken from lean and obese human subjects and failed to observe any effects of body weight on these molecular rhythms^(^ ^25^ ^)^. Although it is possible that differences would have been observed if we had been able to serially sample other (for example, visceral) adipose depots, the data nonetheless indicate that obesity *per se* does not impair clock gene rhythms in all metabolically active tissues.

A small number of animal studies have compared lean and obese groups generated by manipulation of dietary intake, rather than examining the effects of altered body weight due to genetic differences. In most cases, dietary obesity results in clear changes in aspects of the circadian system, but the available results are not entirely consistent. In one study, 6 weeks of high-fat diet altered behavioural and endocrine rhythms, together with changes in gene expression profiles that included reduced amplitude clock gene rhythms in adipose and liver^(^ ^127^ ^)^. In another experiment, mice were fed for 7 weeks with either a high- or low-fat diet, fasted for 24 h and killed in constant darkness. Comparison of clock gene expression in the liver of these animals revealed a phase delay of approximately 3 h with no consistent reduction in rhythm amplitude in the animals fed on a high-fat diet^(^ ^128^ ^)^. Analysis of two daytime time points in mice maintained on high- or low-fat diets for 11 months also revealed altered liver and kidney clock gene expression^(^ ^129^ ^)^. In contrast to the above studies, which all used male mice, few significant differences were observed in liver and adipose clock gene rhythms from female mice fed high- or low-fat diets for 8 weeks^(^ ^130^ ^)^.

The differences in type and magnitude of response observed in mice chronically fed a high-fat diet probably reflect details of experimental design such as sex of the animal, dietary composition and environmental conditions used during the protocol. Sex is an issue of note in regard to human physiology, where sex differences in circadian rhythms^(^ ^131^ ^)^, adiposity^(^ ^132^ ^)^ and nocturnal postprandial response (described below) have been reported. Furthermore, the extreme changes in dietary intake utilised in animal experiments may not accurately represent typical human diets. It is therefore clear that more research is required in this area before the translational consequences are fully understood.

## Relevance to human lifestyle

As would be expected for an emerging research field, the available literature mostly derives from controlled laboratory experiments. However, the biological principles described have far-reaching implications for many people living in contemporary society.

### Dietary regulation of body weight

Evidence is rapidly accumulating to support an important role of meal times in the long-term regulation of body weight. Proof-of-concept studies in animal models have utilised different timed feeding paradigms. Mice housed in a light–dark cycle and fed a high-fat diet gain more weight when the food is available only throughout the light phase, when they would usually be resting, than when it is provided throughout the dark phase^(^ ^133^ ^)^. This body-weight effect becomes statistically significant within 2 weeks and despite trends towards increased energy intake and reduced activity in the light-fed animals, there were no statistically significant changes in these parameters. In a refinement of the protocol, mice were fed a high- or low-fat diet that was available either *ad libitum* throughout the day, or during a 4 h period in the middle of the light phase^(^ ^134^ ^)^. Remarkably, temporal food restriction resulted in reduced body weight on both high- and low-fat diets, to the extent that mice on a restricted high-fat diet weighed less than those provided with a low-fat diet *ad libitum*. This occurred despite no difference in energy consumption (as expressed relative to body weight) between the restricted high-fat-diet group and the two low-fat-diet groups, although mice under restricted feeding did exhibit elevated total daily activity compared with *ad libitum* controls^(^ ^134^ ^)^. Comparing these two studies, it seems that the duration of restricted food availability has profound effects on body-weight regulation, but the mechanisms underlying this phenomenon are not yet clear.

In humans, there is an increasing interest in the effects of meal timing *per se* on metabolism and body weight. Work in this area has understandably focused to a large degree on meals taken at the start and end of the day, for instance the study of breakfast consumption and individuals with night eating disorders. Evidence suggests a role for regular breakfast consumption in the maintenance of healthy body weight, although the issue has rarely been approached from a chronobiological perspective and questions remain about the causative mechanisms involved^(^ ^135^ ^,^ ^136^ ^)^. Night eating syndrome has recently been included in the fifth edition of the Diagnostic and Statistical Manual of Mental Disorders (DSM-5). It is broadly characterised by recurrent episodes of nocturnal eating that cannot be better accounted for by other behavioural and psychiatric disorders, and is discussed in detail elsewhere^(^ ^137^ ^)^. The relationship between night eating syndrome and body weight is complex, with some variable findings reported in the literature. Despite this, the overall evidence provides compelling associations between night eating and obesity, with a consistent finding that night eating syndrome is more prevalent in overweight and obese groups^(^ ^138^ ^)^. The importance of evening meals is further highlighted in studies of subjects without night eating disorder. For example, energy consumption after 20.00 hours has been associated with BMI independently of age, sleep timing and sleep duration^(^ ^139^ ^)^. This group later reported that protein intake within 4 h of sleep onset is associated with elevated BMI after controlling for age, sex, sleep timing and sleep duration^(^ ^140^ ^)^.

The link between food timing and body weight is also apparent in dietary weight-loss studies. A group of 420 individuals undergoing a 20-week weight-loss programme were categorised according to the time at which they ate lunch, which was their main daily meal. Those in the late group lost less weight and at a slower rate than the early group, with no difference in energy intake, energy expenditure, dietary composition or sleep duration^(^ ^141^ ^)^. In a separate experiment, obese/overweight women consumed energy-restricted diets that differed in the proportion of energy distributed between breakfast and dinner. The women eating more energy at breakfast than dinner not only lost more weight but also exhibited an improved metabolic profile in insulin sensitivity and TAG concentration^(^ ^142^ ^)^. Together these data support the hypothesis that the timing of food intake is important for body-weight regulation.

### Shift work and jet-lag

Since industrialisation, humans have gained the ability to regulate environmental conditions and subsequently alter temporal patterns of behaviour. Indeed the phrase ‘24/7 society’ is now in common usage to describe the constant presence of industrial and social activity. In order to cope with the demands of this modern aspect of society, varied work schedules are now commonplace, with approximately 20 % of the European workforce engaged in night shifts^(^ ^143^ ^)^. This clearly indicates that a large section of the population experiences regular misalignment of their behaviour with the solar day. A second common cause of abrupt circadian misalignment is jet-lag, the rapid travel across time zones. Although this experience is a rare and transient phenomenon for most people, it nonetheless affects a substantial number of individuals and in some cases (for example, airline crew) can be a regular event. A related phenomenon that is common in society is a weekly change between widely differing sleep times on work and free days. This has been termed ‘social jet-lag’^(^ ^144^ ^)^ and is associated with elevated BMI^(^ ^145^ ^)^.

Multiple health problems are associated with shift work, including increased risk of cardiovascular and metabolic disease^(^ ^146^ ^)^. Understanding the aetiology of shift work-related morbidity is complex due to diverse contributory factors such as sleep disturbance, altered social pressure and patterns of food intake^(^ ^147^ ^,^ ^148^ ^)^. For example, although shift workers often report normal total energy intake, there is commonly an altered temporal distribution of feeding characterised by more irregular eating times, more snacking and fewer substantial meals^(^ ^148^ ^)^. Alongside these lifestyle changes it is likely that disrupted circadian physiology is a major contributor to the pathophysiological consequences of shift work. Indeed it is generally recognised that many shift workers in temperate regions poorly adapt circadian rhythms to their work conditions^(^ ^149^ ^)^. As a result, these individuals experience prolonged durations of misalignment between their circadian biology and behavioural patterns.

As described previously, postprandial profiles of glucose and TAG concentration vary over the day. Such studies clearly imply that shift workers eat a substantial proportion of their meals during the time of suboptimal glucose and lipid tolerance. This prediction is strengthened by studies of simulated shift work where subjects are subjected to an abrupt shift of typically 6–10 h in their daily routine. Interestingly, the postprandial response in such protocols is altered by the preceding diet. In comparable experiments, the exaggerated postprandial response following a test meal in shifted subjects was reduced for glucose and insulin but increased for TAG following a low-fat pre-meal^(^ ^150^ ^)^ rather than a high-fat pre-meal^(^ ^151^ ^)^. Furthermore, there are reported sex differences in postprandial response, with a more pronounced elevation of TAG in the first night of a simulated night shift in men than in women^(^ ^90^ ^)^.

In real shift workers, there are also postprandial data describing the relative insulin resistance and lipid intolerance following abrupt shift changes^(^ ^152^ ^)^. Given the large number of individuals undertaking shift work in modern society, there is a clear need to improve circadian alignment in these individuals. One possible intervention is the manipulation of light, which is able to reset circadian rhythms and also directly improve alertness^(^ ^153^ ^)^ even following short exposure times^(^ ^154^ ^)^. However, timed food may also be a powerful method for resetting rhythms to a new phase. Of particular interest is the ability of timed food to reset peripheral tissue rhythms.

Animal models support the idea that timed food intake could be a valuable intervention to minimise adverse effects of shift work. Mice subjected to an artificial shift work-like environmental schedule exhibit circadian desynchrony and metabolic disturbance^(^ ^155^ ^)^. Similar findings have been reported in rats exposed to an artificial shift work protocol, although the body-weight increase and metabolic disturbances experienced were attenuated when food availability was restricted to the normal activity phase^(^ ^156^ ^)^. Further development of these animal models will permit detailed molecular analysis to complement human studies of shift work and its adverse effects.

## Conclusion

A wealth of data from varied experimental approaches provides us with clear links between circadian, metabolic and nutritional biology. These findings provide a strong foundation upon which to model mechanisms underlying the temporal differences in response to food intake. One limitation of the field is that little translational research has yet been performed in human subjects. Understanding the circadian regulation of human metabolism will have profound implications for nutritional science and explain how time of day is important for postprandial physiology. Furthermore, it will also reveal how timed dietary intake can be used as a means to alleviate some of the deleterious effects of circadian misalignment that are experienced by large numbers of people within modern society.
